# Single Level Cervical Radiculopathy with and without Fusion Technique: A Systematic Review and Meta-Analysis

**DOI:** 10.1055/s-0046-1820485

**Published:** 2026-07-28

**Authors:** Maria Florencia Deslivia, Sherly Desnita Savio, Benedictus Satmoko, I Gusti Lanang Ngurah Agung Artha Wiguna, Ifran Saleh

**Affiliations:** 1 Bone and Joint Center, St Carolus Hospital, Jakarta, Indonesia; 2 Orthopedics and Traumatology Department, Faculty of Medicine, Udayana University, Prof. I. G. N. G. Ngoerah Hospital, Bali, Indonesia

**Keywords:** cervical vertebrae, decompression, surgical, foraminotomy, meta-analysis, radiculopathy, spinal fusion, artrodese, descompressão cirúrgica, foraminotomia, metanálise, radiculopatia, vértebras cervicais

## Abstract

**Objective:**

To objectively compare the efficacy of single-level cervical radiculopathy decompression with and without fusion, with a focus on randomized controlled trials.

**Methods:**

There were six randomized-controlled studies included in the analysis. Systematic review and meta-analysis were conducted following the Preferred Reporting Items for Systematic Reviews and Meta-Analyses (PRISMA) guidelines. The risk of bias was assessed, data were organized into predefined tables, and calculations were performed using the Review Manager (RevMan) software.

**Results:**

From the analysis of 726 patients (Fusion: 374, Nonfusion: 352), no significant differences were found in kyphotic ( *p* = 0.11), satisfaction ( *p* = 0.73), complication ( *p* = 0.53), recurrence ( *p* = 0.63), or reoperation ( *p* = 0.36) rates. Postoperative pain relief and functional outcomes were comparable between techniques.

**Conclusions:**

Fusion and nonfusion techniques offer comparable efficacy for single-level cervical radiculopathy. However, patient-specific characteristics should be considered. Level of Evidence I.

## Resumo

**Objetivo:**

Comparar objetivamente a eficácia da descompressão para tratamento da radiculopatia cervical de nível único com e sem artrodese, com foco em estudos clínicos randomizados.

**Métodos:**

Esta análise incluiu seis estudos randomizados controlados. Uma revisão sistemática e metanálise foi realizada seguindo as diretrizes Preferred Reporting Items for Systematic Reviews and Meta-Analyses (PRISMA). O risco de viés foi avaliado, os dados foram organizados em tabelas predefinidas e os cálculos foram feitos utilizando o *software* Review Manager (RevMan).

**Resultados:**

A análise de 726 pacientes (artrodese sim: 374 vs. não: 352) não evidenciou diferenças significativas nas taxas de cifose ( *p* = 0,11), satisfação ( *p* = 0,73), complicações ( *p* = 0,53), recidiva ( *p* = 0,63) ou reoperação ( *p* = 0,36). O alívio da dor após a cirurgia e os desfechos funcionais foram semelhantes entre as técnicas.

**Conclusões:**

As técnicas com e sem artrodese apresentaram eficácia comparável para tratamento da radiculopatia cervical de nível único. Entretanto, as características específicas de cada paciente devem ser consideradas. Nível de Evidência: I.

## Key Points

- Nonfusion approaches have demonstrated better cost-effectiveness compared to fusion techniques for cervical radiculopathy, however there has been no consensus yet regarding the most effective treatment for the condition.
- Nonfusion techniques demonstrated comparable kyphotic, satisfaction, complication, recurrence, and reoperation rates, as well as postoperative pain relief, and functional outcomes compared to fusion methods.
- Continued research is essential to refine these techniques and expand the evidence base for informed clinical decision-making.

## Introduction

Cervical radiculopathy, a condition characterized by nerve root compression in the cervical spine, often leads to significant pain, neurological deficits, and impaired quality of life. 1 Surgical intervention is commonly pursued when conservative management fails, with the primary goal of relieving nerve root compression and restoring function. Among the surgical options, anterior cervical discectomy with fusion (ACDF) has been a widely accepted technique, praised for its ability to stabilize the spine postdecompression. 2 3 4

While ACDF has demonstrated favorable outcomes in numerous studies, there are concerns over fusion-related complications and the potential for increased adjacent segment degeneration (ASD), pseudoarthrosis, instrument-related, and ventral approach-related complications. 5 These have led to a growing interest in motion preserving technique, as well as nonfusion approaches, such as anterior cervical discectomy (ACD), 6 posterior foraminotomy (PCF), 7 and posterior endoscopic decompression.

Nonfusion approaches have demonstrated better cost-effectiveness, primarily due to lower direct (surgical instruments and hospitalization) and indirect costs. 8 Previous studies have highlighted the comparative effectiveness of these techniques, 9 10 11 however there has been no consensus yet regarding the most effective treatment for the condition. This systematic review and meta-analysis aims to provide a thorough evaluation of current literature on single-level cervical radiculopathy decompression, with a focus on randomized controlled trials. By objectively comparing clinical outcomes and complications, this review seeks to support clinical decision-making and guide future research in spine surgery.

## Materials and Methods

**Fig. 1:**
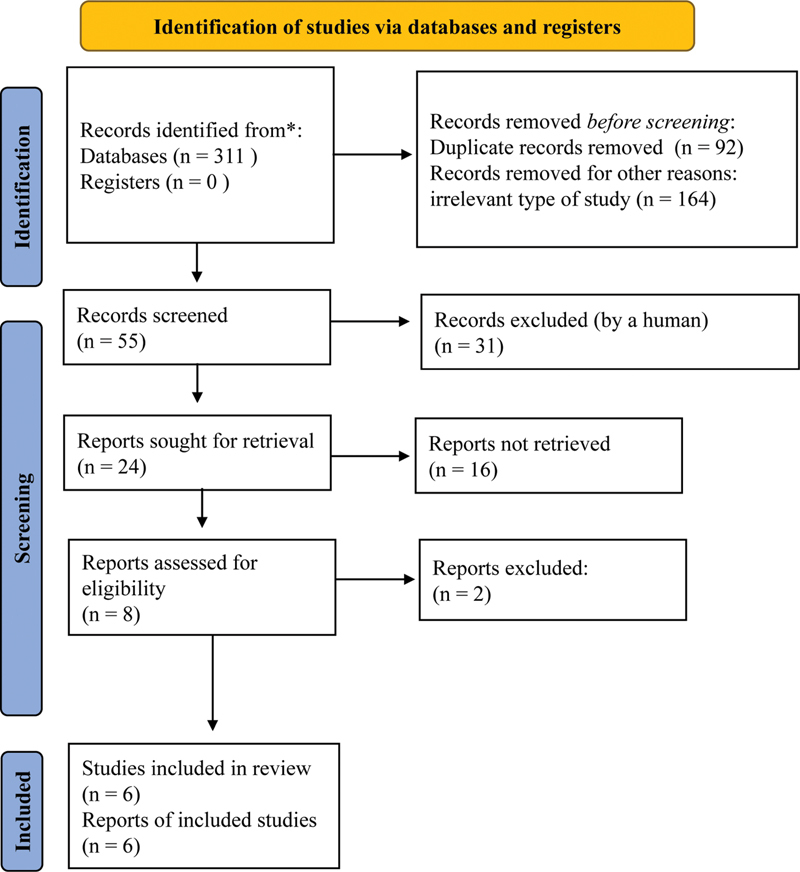
Identification of studies in the primary literature search and the flow diagram of selection process.

This systematic review and meta-analysis were performed in accordance with the Preferred Reporting Items for Systematic Reviews and Meta-Analyzes (PRISMA) guidelines ( Fig. 1 ), as well as the Quality of Reporting of Meta-analyses (QUOROM) checklist and flow diagram for meta-analysis for randomized controlled trials (RCTs). A review protocol was drafted and registered on PROSPERO, number: CRD42024496863.

### Search Strategy and Selection Criteria

We performed a systematic search of English language literature on Google Scholar, PubMed/MEDLINE, Cochrane Central Register of Controlled Trials (CENTRAL), and ClinicalTrials.gov. The final search was conducted on January 15th, 2025. The search strategy combined Medical Subject Headings (MeSH) and free-text keywords related to cervical radiculopathy and surgical techniques, including *cervical radiculopathy* and *anterior cervical discectomy and fusion* , and *randomized controlled trial* . Boolean operators (AND/OR) were applied as appropriate.

The search was filtered to include studies published in English, involving human subjects, and randomized controlled trials. No restrictions on publication year were applied, and only full-text articles were considered eligible. The articles were selected based on the stated inclusion and exclusion criteria according to the Population, Intervention, Comparison, Outcome (PICO) method, as depicted in Table 1 .

**Table 1:** Inclusion and exclusion criteria based on PICO

	Inclusion criteria	Exclusion criteria
**Population**	Adults with single-level cervical radiculopathy post surgical decompression.	Animal studies; Traumatic injury; Reoperations; Underlying congenital condition or neoplasm.
**Intervention**	Decompression techniques without fusion (anterior cervical discectomy, posterior foraminotomy, posterior endoscopic decompression).	Nonoperative procedures; Pharmacologic and nutritional treatment; Standalone physical therapy or rehabilitation.
**Control**	ACDF	
**Outcome**	Clinical outcome: satisfaction, complication, recurrence, pain, functional score; Radiological outcome: kyphotic deformity.	Study is ongoing and no results have been reported; Outcome measures not reported in completion.
**Publication**	Studies published in English in peer-reviewed journals.	Abstracts, editorials, letters; Duplicate publications of the same study that do not report on different outcomes; Meeting presentations or proceedings.
**Study design**	Randomized controlled trials.	Literature review; Noncomparative studies.

### Study Selection and Data Extraction

Study selection was performed independently by two reviewers. Titles and abstracts identified through the database search were screened for eligibility, followed by full-text assessment of potentially relevant articles. Data extraction was also conducted independently by the same two reviewers using a standardized data extraction form. Disagreements at any stage of study selection or data extraction were resolved by consensus; when it could not be reached, a third reviewer was consulted.

The following variables were systematically extracted from each included study: study characteristics (first author, year of publication, study design), patient demographics (sample size, age, sex), surgical characteristics (treated cervical level, type of surgical technique), preoperative neurological status, duration of follow-up, radiological outcomes, clinical outcomes, postoperative complications, recurrence rate, and reoperation rate.

### Quality Assessment

The included RCTs will be assessed in terms of quality by two independent reviewers based on the 2015 Updated Method Guideline for Systematic Reviews 13-items, from the Cochrane Back and Neck Group. 12

Risk of bias assessment was performed to determine studies' eligibility using the Cochrane risk of bias tool in nonrandomized studies for interventions (ROBINS-I) and Risk of Bias 2.0 (RoB 2.0). Domains assessed included the randomization process, deviations from intended interventions, missing outcome data, measurement of outcomes, and selection of the reported results.

### Data Synthesis

From each included study, data related to patient, study characteristics (e.g. age, sex, level treated, and preoperative neurological deficit), and outcomes were extracted and aggregated. Dichotomous variables were assessed in terms of odds ratio (OR) and 95% confidence intervals (CI). Calculations were performed using the Review Manager (RevMan, Nordic Cochrane Centre, Cochrane Collaboration) software, version 5.3. A fixed-effect model was used when heterogeneity (I ^2^ ) was <50%, whereas a random-effect model was used when it was >50%.

## Results

### Literature Search and Study Characteristics

The initial electronic search identified 311 records. After removal of duplicates and screening of titles and abstracts, 8 studies underwent full-text assessment. One study 13 was excluded due to low methodological quality, and another 14 was later excluded because it reported outcomes from the same patient cohort as another included study with a longer follow-up period. 15 Therefore, 6 studies were included in final analysis.

### Baseline Characteristics

A total of 726 patients were analyzed in this study, with more patients in the fusion group (fusion vs. nonfusion: 374 vs. 352). The sample was predominantly male, with the age range of 28 to 67-years-old. The most common level treated C6-7, followed by C5-6. More than half patients had sensory deficit preoperatively in all studies, while 25 to 75% had motoric deficit. The follow-up period ranged from 24 to 69 months ( Table 2 ).

**Table 2:** Sample baseline characteristics of included studies

No	Author (year)	Intervention	Sample size (n)		Gender (Male:Female)		Mean age (years)		Level treated		Neurological deficit		Follow-up (months)
			I	C	I	C	I	C	I	C	I	C	
**1**	Savolainen et al. (1998) 16	Discectomy without fusion	31	ACDF: 30 ACDFI: 30	20:11 (64.5% male)	ACDF: 22:8 (73% male) ACDFI: 21:9 (70% male)	46	ACDF: 47.9 ACDFI: 49.7	C6-7 most common (61.3%), followed by C5-6 (32.26%)	C6-7 most common (46.67%), followed by C5-6 (38.33%)	100% had sensory deficit, 67.74% had motor deficit	78.3% had sensory deficit, 75% had motor deficit	36-60 (average 48)
**2**	Wirth et al. (2000)	PCF ACD without fusion	47	25	26:21 (55.3% male)	14 (56% male)	30-67	28-63	C6-7 most common (48.9%), followed by C5-6 (34%)	C5-6 most common (48%), followed by C6-7 (40%)	78.7% had sensory deficit, 42.55% had motor deficit	84% had sensory deficit, 44% had motor deficit	53-69
**3**	Xie et al. (2007)	Discectomy without fusion	12	ACDF: 15 ACDFI: 15	5:7 (41.67% male)	ACDF: 9:6 (60% male) ACDFI: 14:1 (93.3% male)	42 ± 8	ACDF: 42 ± 8 ACDFI: 43 ± 8	C6-7 most common (58.3%), followed by C5-6 (33.3%)	C5-6 most common (56.67%), followed by C5-6 (36.67%)	58.3% had sensory deficit, 25% had motor deficit	80% had sensory deficit, 36.67% had motor deficit	24
**4**	Ruetten et al. (2008) 17	Full-endoscopic posterior cervical foraminotomy	89	86	Initially 68 (34% male) out of 200 patients		43 (27-62)		C6-7 most common (27.5%), followed by C5-6 (10%)	C6-7 most common (30.5%), followed by C5-6 (11%)	NA		24
**5**	Ruetten et al. (2009) 18	FACD	54	49	Initially 43 (35.8% male) out of 120 patients		30-61		C5-6 most common (53.7%), followed by C6-7 (37%)	C5-6 most common (53%), followed by C6-7 (42.8%)	NA		24
**6**	Broekema et al. (2023) 15	Posterior foraminotomy	119	124	53:66 (45% male)	66:58 (53% male)	51.6 ± 8.5	51 ± 8.3	C6-7 dermatome most common (98%)	Most common dermatome: C7 (50%), followed by C6 (49%)	66% had sensory deficit, 36% had motor deficit	66% had sensory deficit, 41% had motor deficit	24

**Abbreviations:** ACDF, anterior cervical discectomy and fusion; ACDFI, anterior cervical discectomy and fusion with instrumentation; C, control; FACD, full-endoscopic anterior cervical discectomy; I, intervention; NA, not available; PCF, posterior cervical foraminotomy.

### Radiological Outcome

**Fig. 2:**
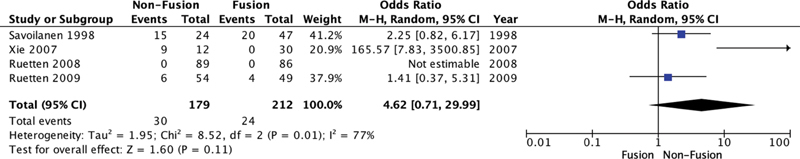
Forest plot for radiological outcome (kyphosis rate). **Abbreviations:** Random, random effects model; df, degrees of freedom; CI, confidence interval; M-H, Mantel-Haenszel.

Tables 3.1 and 3.2 showed the outcome comparison across included studies. Radiological outcome was assessed using kyphotic rate; there was no statistically significant difference between fusion and nonfusion technique for cervical radiculopathy (average difference [AD]: 4.62; 95%CI: 0.71–29.99; I ^2^ = 77%; *p* = 0.11), as shown in Fig. 2 .

**Table 3.1:** Outcome analysis of included studies

No	Author (year)	Radiological (kyphosis rate)		Satisfaction rate		Complication rate		Most common complications
		I	C	I	C	I	C	
**1**	Savolainen et al. (1998) 16	15/24 (62.5%)	20/47 (42.55%)	23/30 (76%)	45/58 (77.6%)	1/31 (3.2%)	56/60 (93.3%)	Severe iliac crest pain in control group (80%)
**2**	Wirth et al. (2000)	NA		NA		NA		NA
**3**	Xie et al. (2007)	9/12 (75%)	0 (0%)	NA		NA		NA
**4**	Ruetten et al. (2008) 17	0 (0%)	0 (0%)	86/89 (96%)	78/86 (91%)	No serious complications. Mild complications in 3/89 (3.3%)	No serious complications. Mild complications in 5/86 (5.8%)	Transient difficulty swallowing in control group (3.5%), transient dermatome-related hypesthesia in intervention group (3%). No serious complications.
**5**	Ruetten et al. (2009) 18	6 (11.8%)	4 (8.3%)	49 (90.7%)	43 (87.8%)	2 (3.7%)	7 (14.3%)	Transient difficulty swallowing in 6.8% (most common in control group), 4% surface hematoma in control group. No serious complications.
**6**	Broekema et al. (2023) 15	NA		70 (73%)	76 (77%)	36 (30%)	35 (28%)	Shoulder symptoms (5.8%), persistent radicular arm pain without need for surgery (4.1%), dysphagia (2.9%), and wound infection (2.9%)

**Abbreviations:** C, control; I, intervention; NA, not available.

**Table 3.2:** Outcome analysis of included studies

		Recurrence rate		Reoperation rate		Postoperative pain		NDI	EQ5D	
**No**	Author (year)	I	C	I	C	I	C	I	C	I	C
**1**	Savolainen et al. (1998) 16	1/31 (3.2%)	1/60 (1.7%)	2/31 (6.5%)	0 (0%)	NA		NA	NA		
**2**	Wirth et al. (2000)	12/47 (25.53%)	8/25 (32%)	9/47 (19.15%)	7/25 (28%)	96% complete relief	100% complete relief	NA		NA	
**3**	Xie et al. (2007)	1/12 (8.3%)	4/30 (13.3%)	NA		Arm pain: 1/12 (8%) Neck pain: 2 (17%)	Arm pain: 1/30 (3.3%) Neck pain: 7/30 (23.3%)	NA		NA	
**4**	Ruetten et al. (2008) 17	3/89 (3.4%)	0 (0%)	6/89 (6.7%)	4/86 (4.7%)	Mean VAS arm: 7 Neck: 16	Mean VAS arm: 8 Neck: 17	NA		NA	
**5**	Ruetten et al. (2009) 18	2 (3.7%)	0 (0%)	4 (7.4%)	2 (6.1%)	Mean VAS Arm: 8 Neck: 15	Mean VAS Arm: 10; Neck: 14	NA		NA	
**6**	Broekema et al. (2023) 15	NA		6 (5%)	4 (3%)	Mean VAS arm: 18.6 ± 22.9; Neck: 24.4 ± 27.5	Mean VAS arm: 15.8 ± 23.7; Neck: 21.7 ± 26.1	17.6 ± 14.6	19.2 ± 16.5	0.84 ± 0.15	0.82 ± 0.14

**Abbreviations:** C, control; EQ-5D, EuroQol five-dimensional questionnaire; I, intervention; NA, not available; NDI, neck disability index; VAS, Visual Analog Scale.

### Clinical Outcome and Patient Satisfaction

**Fig. 3:**
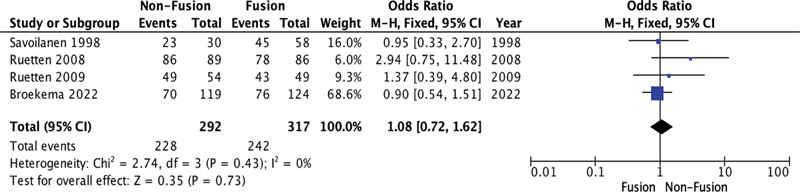
Forest plot for satisfaction rate. **Abbreviations:** Fixed, fixed effects model; df, degrees of freedom; CI, confidence interval; M-H, Mantel-Haenszel.

Satisfaction rate was comparable between the two techniques (AD: 1.08; 95%CI: 0.72–1.62; I ^2^ = 0%; *p* = 0.73), as shown in Fig. 3 .

### Complications

**Fig. 4:**
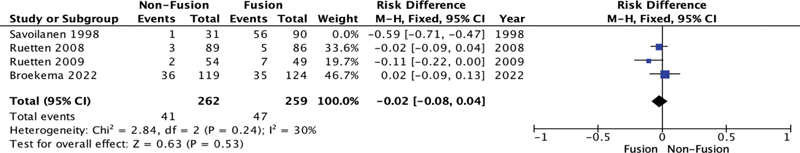
Forest plot for complication rate. **Abbreviations:** Fixed, fixed effects model; df, degrees of freedom; CI, confidence interval; M-H, Mantel-Haenszel.

Postoperative complications were reported across studies. In the fusion group, donor-site morbidity related to iliac crest bone graft harvesting was reported in one study. 16 Initial pooled analysis demonstrated high heterogeneity (I ^2^ = 96%). After exclusion of this study, a decrease was noted (I ^2^ = 30%), with no statistically significant difference in complication rates between fusion and nonfusion techniques (AD: -0.02; 95%CI: -0.08 to 0.04; *p* = 0.53), as shown in Fig. 4 . Other reported complications included transient dysphagia, hematoma, radicular arm pain, and wound infection.

### Recurrence and Reoperation Rate

**Fig. 5:**
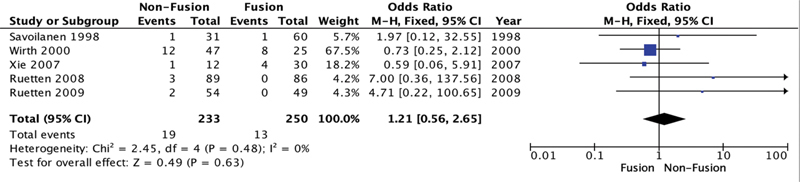
Forest plot for recurrence rate. **Abbreviations:** Fixed, fixed effects model; df, degrees of freedom; CI, confidence interval; M-H, Mantel-Haenszel.

There were no statistically significant differences between fusion and nonfusion techniques in recurrence rate ( *p* = 0.63) or reoperation rate ( *p* = 0.36), as shown in Fig. 5 .

### Functional Outcomes

Functional outcomes assessed using the Neck Disability Index (NDI) and EuroQol five-dimensional questionnaire (EQ-5D) were reported in one study. No statistically significant differences between fusion and nonfusion techniques were observed for these outcomes.

## Discussion

This is the first study to synthesize high-level evidence on recent advancements in single-level cervical radiculopathy management. Analysis of six RCTs revealed that nonfusion techniques—including discectomy, posterior cervical foraminotomy (PCF), and ACD—demonstrate comparable outcomes to fusion techniques in terms of kyphosis rates, patient satisfaction, complications, recurrence, and reoperation rates. However, further exploration of these findings is warranted to provide greater context and clinical insight.

### Clinical Outcome

The ACDF is widely regarded as the “gold standard” for managing cervical radiculopathy due to its well-documented good to excellent clinical outcomes. However, studies by Ruetten et al. 17 18 indicate that nonfusion approaches, such as PCF, achieve similar significant and sustained improvements. Notably, hospitalization time was shorter with nonfusion techniques (3 vs. 7 days for fusion), likely due to less invasive surgical approaches resulting in reduced soft tissue damage.

Several nonrandomized studies have reported that PCF are comparable or even superior to those of ACDF. 19 20 However, the lack of randomization introduces potential selection bias, as PCF may have been preferentially chosen for patients with more favorable pathology, such as soft, lateral disc herniations or preserved disc height, which are inherently more responsive to decompression alone. Another study comparing percutaneous endoscopic cervical discectomy (PECD) and ACDF reported slightly higher revision rates in the PECD group. 21 Notably, PECD procedures were performed by expert endoscopic surgeons, while ACDFs were performed by a broader group with varying levels of experience. These technical and methodological factors may introduce bias in favor of nonfusion techniques and limit the generalizability of the findings.

To minimize the bias in nonrandomized designs, this meta-analysis focuses primarily on RCTs. However, despite the strength of this design, several methodological limitations remain that may introduce bias in favor of nonfusion approaches. First, patients and surgeons were not blinded to the assigned intervention, which raises the risk of performance and detection bias, particularly in subjective outcomes like satisfaction and return to work. Nonfusion procedures often are associated with smaller incisions and less tissue disruption, which could inherently reduce complication rates and enhance patient perception.

Additionally, potential selection bias may have occurred during patient enrollment. Some surgeons expressed a preference for one of the treatment arms, primarily favoring ACDF, which may have influenced eligibility decisions. 15 Second, the procedure was performed without anterior plating, using standalone polyetheretherketone (PEEK) cages. 14 15 While this reflects certain regional practices, the lack of plating may compromise segmental stability and fusion rates, potentially underrepresenting the efficacy of ACDF. Nevertheless, previous meta-analyses 22 have suggested that the outcomes of cage-only constructs may not be inferior to plated ones, though this remains context-dependent.

Radiological outcomes, including kyphotic alignment, did not demonstrate clinically meaningful differences between fusion and nonfusion techniques. Similarly, functional measures such as the NDI and visual analogue scales (VAS) for neck and arm pain showed substantial postoperative improvement across both approaches. Beyond these objective metrics, patient-reported outcomes (quality of life, return-to-work, and overall satisfaction) were similarly favorable. 19 23 This underscores the importance of individualized treatment planning, based on patient preferences and specific clinical presentations.

### Complications, Recurrences, and Reoperations

While complication rates between fusion and nonfusion groups were similar, the nature of these complications differed. Fusion procedures frequently reported postoperative dysphagia, hematoma, and transient unilateral recurrent laryngeal nerve palsy. Conversely, nonfusion techniques exhibited higher rates of surface hematoma and complications such as frozen shoulder and supraspinatus tendinitis, particularly with PCF. 14 15 Reoperations at the index level and wound infections were also more prevalent in nonfusion procedures, underscoring the importance of careful surgical technique and patient selection.

Lin et al. 23 observed that while both ACDF and PCF resulted in marked pain relief, the nonfusion group experienced a higher incidence of secondary procedures related to restenosis. MacDowall et al. 24 further highlighted that although both groups achieved significant reductions in NDI scores over a 5-year period, the reoperation rate was more pronounced in the nonfusion group (approaching 1/10) compared to the fusion group (about 1/25). Foster et al. 25 also supported these findings by showing comparable outcomes between PCF and ACD, with minimal differences in reoperation rates. Persistent radicular arm pain, despite not requiring surgery, was more common in nonfusion patients. This observation raises concerns about the long-term efficacy of nonfusion techniques and highlights the need for further research to mitigate residual symptoms.

While this study focused primarily on immediate and short-term outcomes, the long-term implications, such as ASD, warrant further investigation. Fusion procedures are associated with an increased risk of ASD due to altered biomechanical loading, a complication that appears less common in nonfusion techniques. Future studies should aim to determine whether nonfusion approaches reduce ASD incidence and related complications over extended follow-up periods.

## Future Considerations

The cost-effectiveness of surgical options is an increasingly critical consideration. Nonfusion techniques may lower healthcare costs through shorter hospital stays and faster recovery. 9 12 However, the potential for higher reoperation rates must also be factored into economic evaluations. Additionally, tailored postoperative rehabilitation protocols could further optimize outcomes and reduce complications for both fusion and nonfusion procedures, warranting dedicated exploration in future studies.

Innovative minimally invasive procedures are emerging as promising alternatives for the treatment of cervical radiculopathy. Comparative studies, including those by Dunn et al. 20 and Ahn et al., 21 indicate that these emerging techniques can achieve clinical outcomes that are equivalent to those of traditional ACDF. Nonetheless, gaps in the current evidence base emphasize the need for more robust, high-quality studies with standardized outcome measures and longer follow-up durations to refine surgical strategies further.

## Limitations

This meta-analysis has several limitations. First, the number of included studies per outcome was limited, with some key clinical endpoints (e.g., NDI, EQ-5D, and kyphosis) reported in only a few trials. As a result, pooled estimates for these outcomes are based on sparse data and subject to considerable heterogeneity, which reduces the certainty of the findings. Therefore, these conclusions should be interpreted with caution.

Second, the nonfusion group comprised heterogeneous surgical techniques, including anterior discectomy without fusion, posterior foraminotomy, and endoscopic anterior or posterior decompression. Although subgroup analyses according to specific nonfusion techniques were considered, the limited number of studies and inconsistent outcome reporting precluded meaningful subgroup comparisons. Consequently, all nonfusion procedures were pooled for analysis. This clinical heterogeneity may have diluted true technique-specific effects, contributed to increased statistical heterogeneity, and limited the interpretability of the pooled effect estimates, which should be regarded as an average effect across diverse nonfusion procedures rather than a comparison with a single surgical technique.

Third, due to the nature of surgical interventions, blinding of both surgeons and patients was not feasible in any of the included trials, which introduces an inherent risk of bias.

Nevertheless, the studies were rigorously designed, most with independent outcome assessors and consistent follow-up protocols. Furthermore, surgical trials with adequate randomization, intention-to-treat analysis, and objective outcome measures, such as reoperation rate and complication profile, still offer valuable and clinically relevant evidence. Despite the unavoidable limitations of surgical RCTs, these studies remain the highest available level of evidence to guide decision-making between fusion and nonfusion techniques in single-level cervical radiculopathy.

## Conclusion

This study highlights the comparable efficacy of fusion and nonfusion techniques in managing single-level cervical radiculopathy, each with distinct advantages and limitations. By considering patient-specific factors, surgical innovations, and long-term outcomes, clinicians can better tailor interventions to achieve optimal results. Continued research is essential to refine these techniques and expand the evidence base for informed clinical decision-making.
